# Rapid structural remodeling of peripheral taste neurons is independent of taste cell turnover

**DOI:** 10.1371/journal.pbio.3002271

**Published:** 2023-08-31

**Authors:** Zachary D. Whiddon, Jaleia B. Marshall, David C. Alston, Aaron W. McGee, Robin F. Krimm

**Affiliations:** Department of Anatomical Sciences and Neurobiology, University of Louisville School of Medicine, Louisville, Kentucky, United States of America; Universitat Regensburg, GERMANY

## Abstract

Taste bud cells are constantly replaced in taste buds as old cells die and new cells migrate into the bud. The perception of taste relies on new taste bud cells integrating with existing neural circuitry, yet how these new cells connect with a taste ganglion neuron is unknown. Do taste ganglion neurons remodel to accommodate taste bud cell renewal? If so, how much of the structure of taste axons is fixed and how much remodels? Here, we measured the motility and branching of individual taste arbors (the portion of the axon innervating taste buds) in mice over time with two-photon in vivo microscopy. Terminal branches of taste arbors continuously and rapidly remodel within the taste bud. This remodeling is faster than predicted by taste bud cell renewal, with terminal branches added and lost concurrently. Surprisingly, blocking entry of new taste bud cells with chemotherapeutic agents revealed that remodeling of the terminal branches on taste arbors does not rely on the renewal of taste bud cells. Although terminal branch remodeling was fast and intrinsically controlled, no new arbors were added to taste buds, and few were lost over 100 days. Taste ganglion neurons maintain a stable number of arbors that are each capable of high-speed remodeling. We propose that terminal branch plasticity permits arbors to locate new taste bud cells, while stability of arbor number supports constancy in the degree of connectivity and function for each neuron over time.

## Abstract 2

Taste bud cells are constantly replaced in taste buds as old cells die and new cells migrate into the bud, but how do these new cells connect with a taste ganglion neuron? This study uses in vivo two-photon microscopy to observe motility and branching of taste axon arbors, revealing remarkable structural plasticity in taste buds.

## Introduction

The replacement of cells in the taste bud was discovered more than 50 years ago [1]. In fact, cells within a taste bud are one of the fastest renewing cell populations in the body, with an average life span of 10 days [1–4]. Taste bud cells transduce chemical stimuli in the oral cavity and transmit a signal to the axons of taste ganglion neurons, which carry this information to the brain. Peripheral taste neurons are like other pseudounipolar sensory neurons in that each has a single process (axon) projecting from the cell body with an axon hillock. The single axon bifurcates and has a central terminal and peripheral receptive process. Because the peripheral portion of the taste axon is connected to taste bud cells, each time a taste bud cell dies, the axon of a taste ganglion neuron must connect with a new taste bud cell. These continuous reconnections are required if the system is to maintain functional integrity over time [5–7]. How this process is orchestrated is still unclear but could involve structural remodeling of the peripheral taste axon [8,9]. Alternatively, taste bud cells could migrate to the peripheral axon of the taste ganglion neuron, making neuronal remodeling unnecessary.

Progress toward understanding the cellular movements occurring during taste bud cell turnover has been hindered by analysis of fixed-tissue [1,2,10,11]. This approach fails to capture the dynamic processes within the taste bud, such as taste cell movements and axon remodeling [1,2,12]. Fixed-tissue studies also fail to describe what happens to axon remodeling when cell turnover is disrupted (e.g., chemotherapies) [6,8,13]. Additionally, because it is unknown whether taste ganglion neurons undergo structural remodeling, it is unclear why there are morphological differences between individual taste ganglion neurons [14]. Morphological differences between individual taste ganglion neurons could be inherent features of the neuron that dictate differences in functional properties or could be snapshots in time of a continuously remodeling circuit that lacks functional stability. Understanding how taste ganglion neuron structure changes over time is required to relate taste ganglion neuron morphology to function in the taste system.

In other parts of the nervous system, longitudinal in vivo imaging has been used to study the structural plasticity of axons and dendrites over time. Structural remodeling has been shown to occur during development and following injury or disease [15–21]. Under these conditions, axonal degeneration is the process that has been most extensively described [22,23]. In adult neurons, dendritic branches are stable and remodeling is restricted to dendritic spine turnover [24–26]. Axons may show a greater capacity to remodel than dendrites, but the magnitude of this remodeling varies between types of neurons [27]. It has not been determined if peripheral taste axons have the capacity to remodel and, if so, to what extent remodeling occurs.

Here, we examined the structure of peripheral taste axons using in vivo two-photon microscopy to determine how the structure of taste ganglion neurons changes over time. We discovered that peripheral axons of taste ganglion neurons have regions that rapidly remodel and regions that are stable throughout adulthood. Terminal branches (branch produced by the most peripheral branch point in the arbor) remodel rapidly, faster than expected based on the rates of taste cell turnover. This structural plasticity appears to be an inherent feature of the neurons and is not regulated by cell turnover. The total number of arbors (the portion of the axon that innervates the taste bud) for each taste ganglion neuron is stable. We conclude that taste ganglion neurons maintain their peripheral circuitry by having a stable number of arbors, with each arbor continuously remodeling to connect with new taste bud cells over time. We propose this combination of stability and plasticity supports consistency in the total number of taste-transducing cells providing input to an individual taste ganglion neuron and provides flexibility for forming connections with a continuously renewing population of taste cells.

## Results

### Acute in vivo imaging reveals axonal structural plasticity

**Fig 1:**
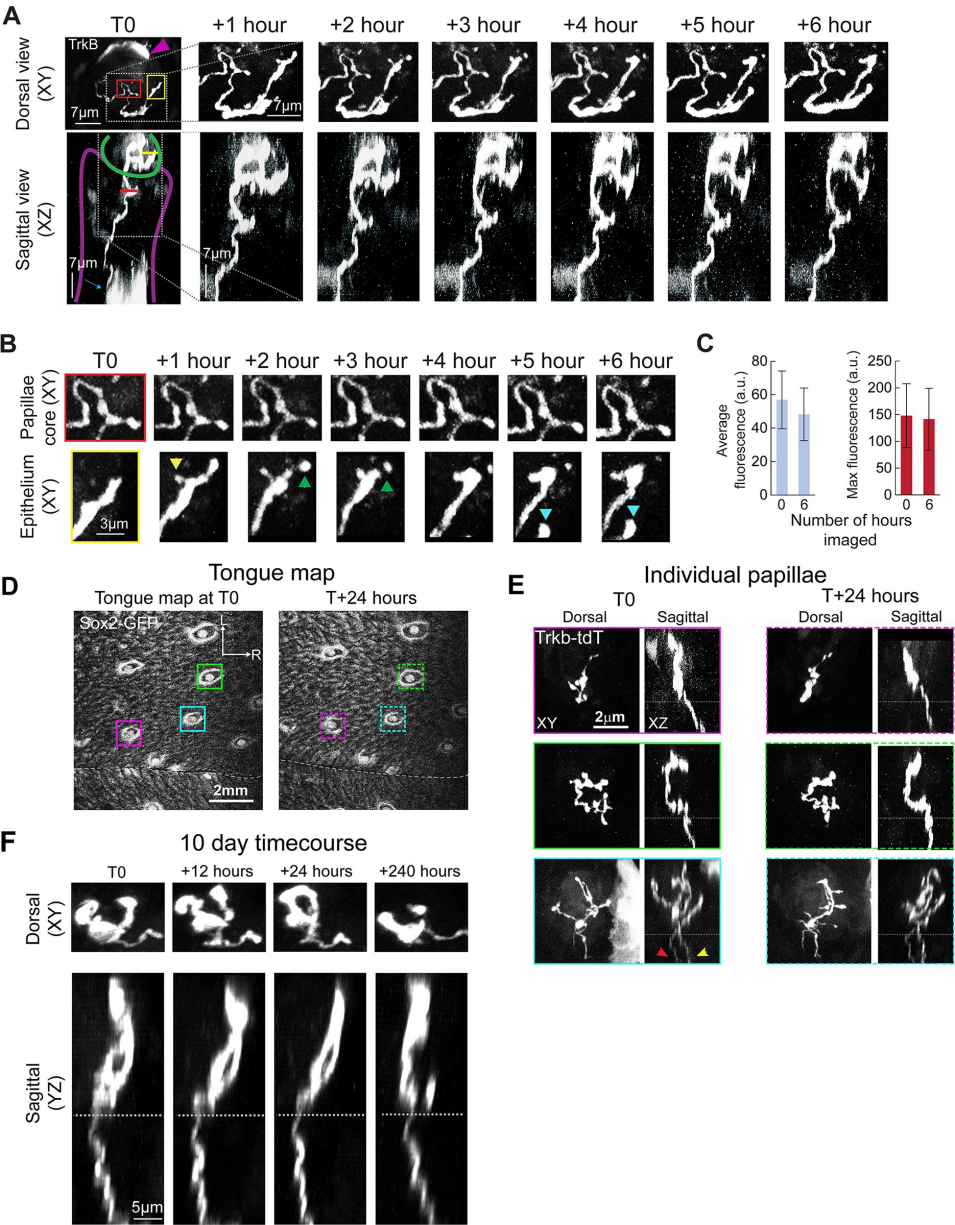
Intravital imaging of taste neurons in fungiform papillae. An example of a fungiform papilla with a single tdTomato-labeled receptive arbor illustrating small-scale changes within 6 hours. (**A**) Top row shows the z-projection of an arbor imaged parallel to the dorsal tongue surface (X, Y). Bottom (XZ) shows a Y projection view of the same arbor within a fungiform papilla. This arbor was imaged every hour over a 6-hour period. Some keratinocytes were also labeled (magenta arrowhead; [33]). After T0, images for each consecutive time point are enlarged to show only the labeled arbor. At T0, the taste bud is outlined (green line), and the connective tissue core of the papilla is outlined (purple line). Background fluorescence levels permitted the regions of the papilla to be distinguished. (**B**) High-magnification view (XY projection) of the arbor within the papilla core (red box) and taste bud (yellow box). The portion of the branch ending inside the taste bud adds a small process (yellow arrowhead) after 1 hour and then retracts a small process (green arrowhead) resulting in a change in branch end shape. Another branch grows into view at hour 5 (cyan arrowhead). The portion of the axon in the papilla core did not change across 6 hours. (**C**) Comparison of average and maximum fluorescence (mean ±SD), of the same receptive arbors at hour 0 and hour 6, from an experiment in which arbors were imaged every hour for 6 hours. Fluorescence levels between hour 0 and 6 were unchanged. (**D**) GFP expression in the anterior tongue epithelium illustrating a papillae map. Papillae with labeled arbors are indicated by solid boxes at T0 and dashed boxes 24 hours later. The tongue midline is indicated with a white dashed line, and rostral (R) and lateral (L) directions are indicated by white arrows. (**E**) High-magnification micrographs of TrkB-tdTomato-labeled nerve arbors in each of the 3 papillae indicated from the map. The first column shows the XY (dorsal) plane, which is parallel to the tongue surface, and the YZ plane is shown adjacent to XY. The papillae boxed in magenta and green had a single labeled arbor, and the papilla boxed in cyan had 2 separate labeled arbors, which are best visualized by counting the axons below the taste bud (yellow and red arrows). Micrographs outlined in dashed lines show high-magnification views of the same papillae 24 hours later. Some changes to each arbor can be seen during this time within the taste bud but not within the papillae core. (**F**) An example arbor is shown at the initial time point (T0) and 12, 24, and 240 hours later. Gray dashed lines in the z-plane images illustrate the approximate base of the taste bud defined by the background labeling produced by GFP expression, although GFP was not imaged separately to reduce laser exposure. The data underlying the graphs in the figure can be found in https://data.mendeley.com/datasets/d58vz7wfrf/1.

To determine whether the structure of the peripheral taste axon is fixed or remodels over time, we developed an in vivo preparation to image the portion of the axon of a taste ganglion neuron that terminates in the taste bud (S1 Fig, arbors). These arbors enter taste buds at the basement membrane of the lingual epithelium. On the anterior two-thirds of the mammalian tongue, taste buds are housed in epithelial structures called fungiform papillae, which are easily accessible for imaging in live animals [28]. Within taste buds, arbors form a dense plexus with many overlapping branches [11,29]. We used a sparse-labeling strategy to express tdTomato in a small number of taste ganglion neurons so that individual arbors could be distinguished [14]. In this preparation, fewer than 5 arbors were labeled with tdTomato per fungiform taste bud and many taste buds did not contain any labeled arbors. Individual arbors were imaged using two-photon laser scanning microscopy (Fig 1A).

We found that individual arbors could be imaged to a depth of 70 μm to capture the entire arbor, and a portion of the axon beneath the taste bud (S1 Fig). To estimate the frequency of structural plasticity for these arbors, first, we imaged the same arbors once per hour for a 6-hour period (*n* = 36). We found that the structure of the axon within the lamina propria (Fig 1A, purple line) was stable (Fig 1B, red box). Many of the terminal branches of arbors (Fig 1A, green line) displayed structural changes as quickly as within a few hours after the initial imaging session (Fig 1B, yellow box). Among the 36 arbors imaged, 24 arbors changed their number of terminal branches in a 6-hour window by adding and/or subtracting small terminal branches (Fig 1B, yellow box).

To determine whether the low level of excitation power (see Methods) used for imaging induced evident photobleaching, we imaged a new group of arbors (*n* = 13 from 2 mice) using identical acquisition parameters every hour for 6 hours and compared the fluorescence intensity across image stacks (z-max intensity projections) for the first and last time points. We did not observe a significant decrease in average (t(24) = 1.34, *p* = 0.76) or maximum (t(24) = 0.27, *p* = 0.78) fluorescence intensity despite imaging these structures repeatedly (Fig 1C). Thus, this imaging approach was suitable for acute in vivo imaging of individual arbors and demonstrates that arbors of taste ganglion neurons display high-speed structural remodeling.

### Chronic in vivo imaging requires a fungiform papilla map

Next, we examined axonal structural plasticity over days. Reliably identifying the same taste buds across imaging sessions was critical for chronic imaging experiments. To identify the same taste buds over days or weeks, we combined our approach for sparse labeling of taste ganglion neurons (*Ntrk2*^CreER^:tdTomato) with transgenic mice that express green fluorescent protein (GFP) in taste buds (*Sox2*^GFP^; [30]). When viewed at low magnification, GFP expression in the anterior tongue epithelium revealed a unique taste bud pattern for each mouse (Fig 1D). This provided a set of fiduciary marks used to locate the same taste buds and arbors repeatedly over time. Three taste buds in the example papillae map contained labeled arbors; they are shown at high magnification 24 hours apart (Fig 1E). No other fungiform taste buds viewed in this field contained labeled arbors due to the sparseness of genetic labeling of taste ganglion neurons (Fig 1D, taste buds with no box).

Taste bud cells have an average life span of 10 days [1]. Given this rate of taste bud cell turnover, we investigated the structural plasticity of arbors every 12 hours for 10 days. In 5 mice, we observed the same 31 taste buds and 60 labeled arbors 21 times over 10 days. We have made these image stacks publicly available (10.6084/m9.figshare.23589351). Among these arbors, 4 were lost during this period, and none were added. One taste bud lost GFP expression in the epithelium, but the arbor remained, consistent with taste bud loss as the animal ages [31,32]. Examination of a single-labeled arbor at the beginning and end of the 10-day experiment illustrates that portion of the axon in the papillae core was stable, whereas the arbor structure in the taste bud was plastic (Fig 1F).

### Terminal branches remodel rapidly and continuously over 10 days

The arbors of taste ganglion neurons connect with taste bud cells [10,11,34,35]. These connections are classical synapses or CALHM1/3 channels [34,36,37]. Unfortunately, there is not a specific anatomical trait, for example, terminal varicosities, in the arbor that have been described in conjunction with these connections. Contacts between taste arbors and taste bud cells frequently occur on the terminal branches of arbors [12]. If the turnover of taste bud cells regulates arbor structure, then terminal branches are likely to remodel as taste bud cells die and/or new cells enter the taste bud. Arbors form synapses with an average of 1.6 taste bud cells [10]. If new terminal branches are added and lost as a means of synapsing with new taste bud cells, then most arbors should add or lose roughly 1 terminal branch over 10 days, and a few arbors should remain stable.

**Fig 2:**
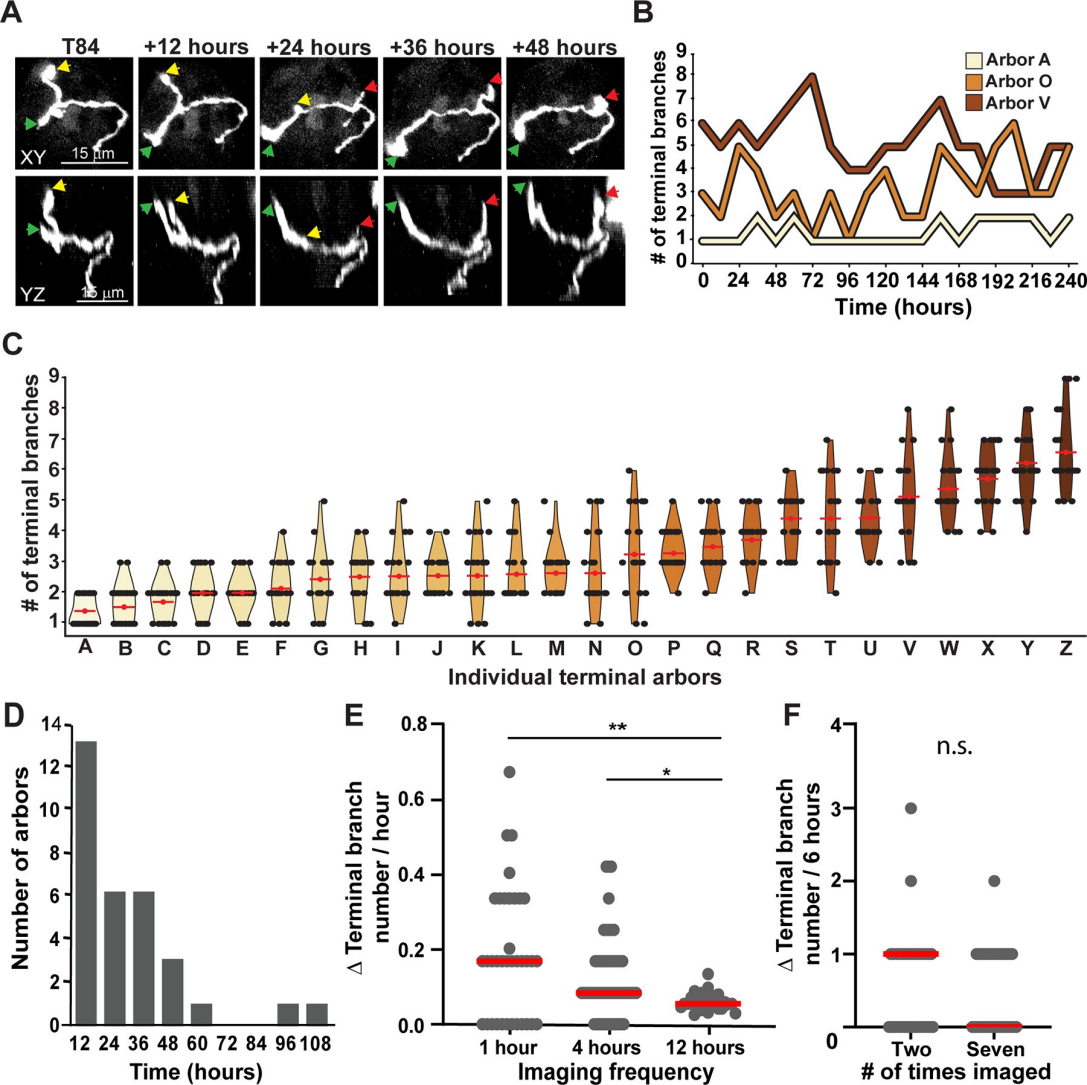
Terminal branches are continuously and rapidly remodeled. (**A**) Example arbor shown every 12 hours over 2 days (for the full 10 days, see S1 Movie). Terminal branches labeled with green and red arrowheads extended during this period, while the terminal branch labeled with a yellow arrowhead retracted. (**B**) The number of terminal branches every 12 hours for 3 different arbors across the 10-day imaging window. (**C**) Violin plots for 26 individual arbors (A-Z) presenting showing the distribution of terminal branch number during the 10-day imaging window. Arbors are arranged in ascending order of average terminal branch number (red dot and bar) across 10 days. (**D**) Amount of time until the first gain or loss of a terminal branch. All arbors added or lost a terminal branch within 108 hours. (**E**) Plots compare rates of change in terminal branch number per hour for 3 different sampling frequencies: 1 hour (*n* = 4), 4 hours (*n* = 4), or 12 hours (*n* = 5); red bars indicate median value (single asterisk *p* < 0.05, double *p* < 0.001). (**F**) Rate of change in terminal branches over 6 hours for arbors imaged twice compared with arbors imaged 7 times (U = 465, *p* = 0.61); red bars indicate median value. The data underlying the graphs in the figure can be found in https://data.mendeley.com/datasets/d58vz7wfrf/1.

In 5 mice, we imaged 31 taste buds containing a total of 60 arbors. However, taste buds with 3 or more labeled arbors were not quantified because we could not distinguish arbors as separate when branches intermingled. We identified 26 individual arbors from 21 different taste buds that were distinguishable at all time points and quantified their terminal branches every 12 hours (Fig 2A and 2B and S1 Movie). All quantified arbors added and lost multiple terminal branches within 10 days (Fig 2B and 2C). In 43% of arbors, a terminal branch was added or lost within the first 12 hours, and 100% of arbors gained or lost a branch within the first 4.5 days (108 hours; Fig 2D). For the 30 arbors imaged every 12 hours for at least 4 days, 1 terminal branch was added or lost approximately every 17 hours (Fig 2E). This anatomical plasticity was much faster than predicted based on the rate of taste bud cell turnover. Thus, terminal branches do not appear to be added for the primary purpose of synapsing with a new taste bud cell or lost solely as a mechanism for disconnecting from dying taste bud cells.

**Fig 3:**
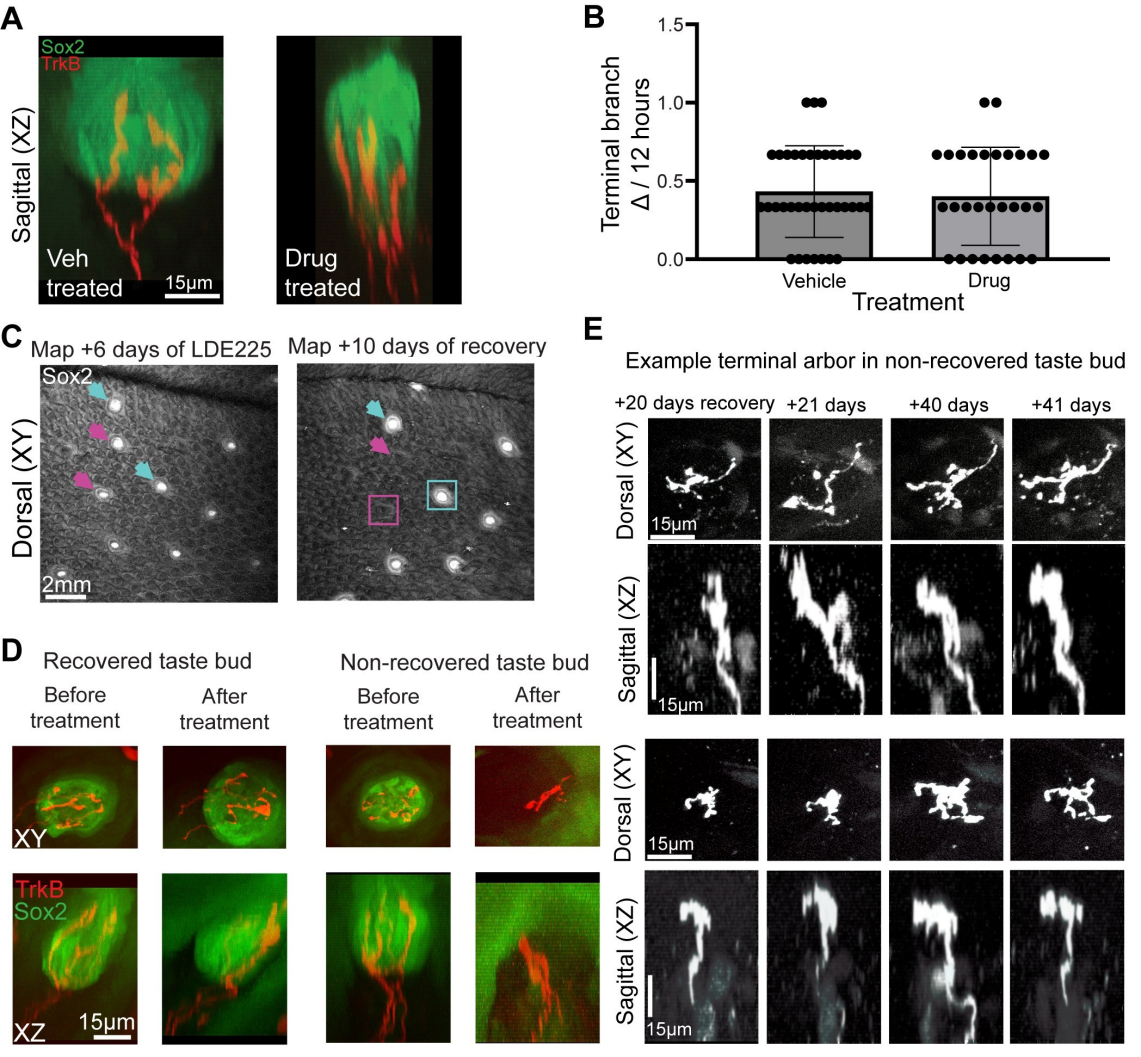
Terminal branch remodeling is not influenced by addition of new cells into the taste bud. (**A**) Example vehicle-treated and drug-treated taste buds after 8 days of treatment. (**B**) There was no difference in the rate with which terminal branches were gained/lost when taste bud cell turnover was halted (LDE225-treated mice, 37 arbors from 6 mice) compared with normal conditions (vehicle-treated/untreated mice, 29 arbors from 5 mice). (**C**) After LDE225 treatment, some fungiform papillae did not regain taste buds as determined by GFP (magenta arrows and box). Two recovered taste buds are indicated with blue arrows and box. (**D**) Before and after images of individual fungiform papillae with a taste bud had high levels of GFP (recovered), and one that lacked GFP expression (nonrecovered) 20 days after LDE225 treatment. Papillae with both recovered and nonrecovered taste buds retained labeled arbors. (**E**) Two papillae containing nonrecovered taste buds with single-labeled arbors are shown 20, 21, 40, and 41 days after LDE225 treatment (*n* = 6 mice). Both arbors continued to remodel somewhat despite the absence of a taste bud. The data underlying the graphs in the figure can be found in https://data.mendeley.com/datasets/d58vz7wfrf/1.

Given the high rate of terminal branch addition and removal, we suspected that we could be underestimating the rate of change if some terminal branches were both gained and lost within the 12-hour intervals between imaging sessions. To test this possibility, we imaged arbors every 4 hours for a 12-hour period. An average of 1 terminal branch was added or lost every 8 hours (*n* = 57 arbors), which was a significantly faster rate of change than the one obtained when arbors were imaged every 12 hours (Fig 3E, *p* < 0.02, Kruskal–Wallis). These results indicate that imaging every 12 hours underestimates the rate of terminal branch change. To verify the accuracy of this rate of terminal branch change, we compared arbors imaged every 4 hours to arbors imaged every hour (*n* = 36) and found no significant difference in the rate of terminal branch gain/loss (1-hour interval versus 4-hour interval, *p* = 0.96 (Kruskal–Wallis); Fig 2E). Thus, we conclude that accurately describing changes in terminal branch number requires sampling the arbor every 4 hours. However, given the high rate of terminal branch addition and loss, we also sought to determine whether two-photon exposure influences the rate of terminal branch addition/loss. We compared arbors imaged twice, 6 hours apart, with arbors imaged 7 times over the same amount of time (Fig 2F). There was no difference in the rate of change in terminal branch number over the 6-hour period between these 2 sets of arbors (*p* = 0.61, Mann–Whitney), indicating that image collection does not influence the rate at which terminal branches are added and lost.

### Gain/loss of terminal branches is not regulated by taste bud cell turnover

If arbors add new terminal branches to synapse with new taste bud cells, then altering the rate of new cell entry would be expected to alter terminal branch structural plasticity. To test the hypothesis that taste ganglion neuron remodeling is dependent on the addition of new taste bud cells, we prevented new cells from entering taste buds by blocking Hh-signaling. Administering LDE225 (trade name Sonidegib) stops new taste cells from differentiating and entering taste buds [6,13,38–41] (S2 Fig). This chemotherapeutic agent is used to treat basal cell carcinoma and is associated with loss of taste by patients receiving treatment [13,42,43]. Over time, old taste cells continue to die and taste bud volume decreases [39] (S2 Fig).

We treated mice with LDE225 (*n* = 4) or vehicle (*n* = 2) for 10 days and imaged taste arbors every 4 hours for 12 hours on days 6, 8, and 10; this was required since a longer imaging window would fail to capture all the terminal branch additions and losses (Fig 2E). Despite the prevention of new cell entry into the taste bud, arbors remained in the taste buds of mice treated with LDE225 and appeared similar to arbors from vehicle-treated taste buds (Fig 3A). Because arbors had similar rates of terminal branch gain/loss on each day of imaging (H(30) = 29.5, *p* = 0.44), the data were combined across days. We found that the rate of terminal branch gain/loss was similar between the LDE225 and vehicle groups (U = 312, *p* = 0.61) (Fig 3B). Thus, we conclude that preventing new cells from entering taste buds does not alter the rate of terminal branch remodeling.

We also examined how taste buds recover following treatment with LDE255. Of 33 imaged taste buds, 11 recovered normal taste bud morphology; however, 22 failed to recover and lacked Sox2 expression up to 40 days after daily LDE225 treatment for 10 days (Fig 3C). Nonrecovered papillae (no GFP expression) retained arbors even in the absence of taste buds (Fig 3D). Surprisingly, we observed at least some remodeling of terminal branches in all papillae that lacked taste buds over 20 days, although remodeling over 24 hours was limited (Fig 3E). We conclude from these findings that the taste bud is not required for terminal branch remodeling of taste arbors, although the rate of remodeling may be slowed.

### Growth and retraction occur concurrently within the arbor

**Fig 4:**
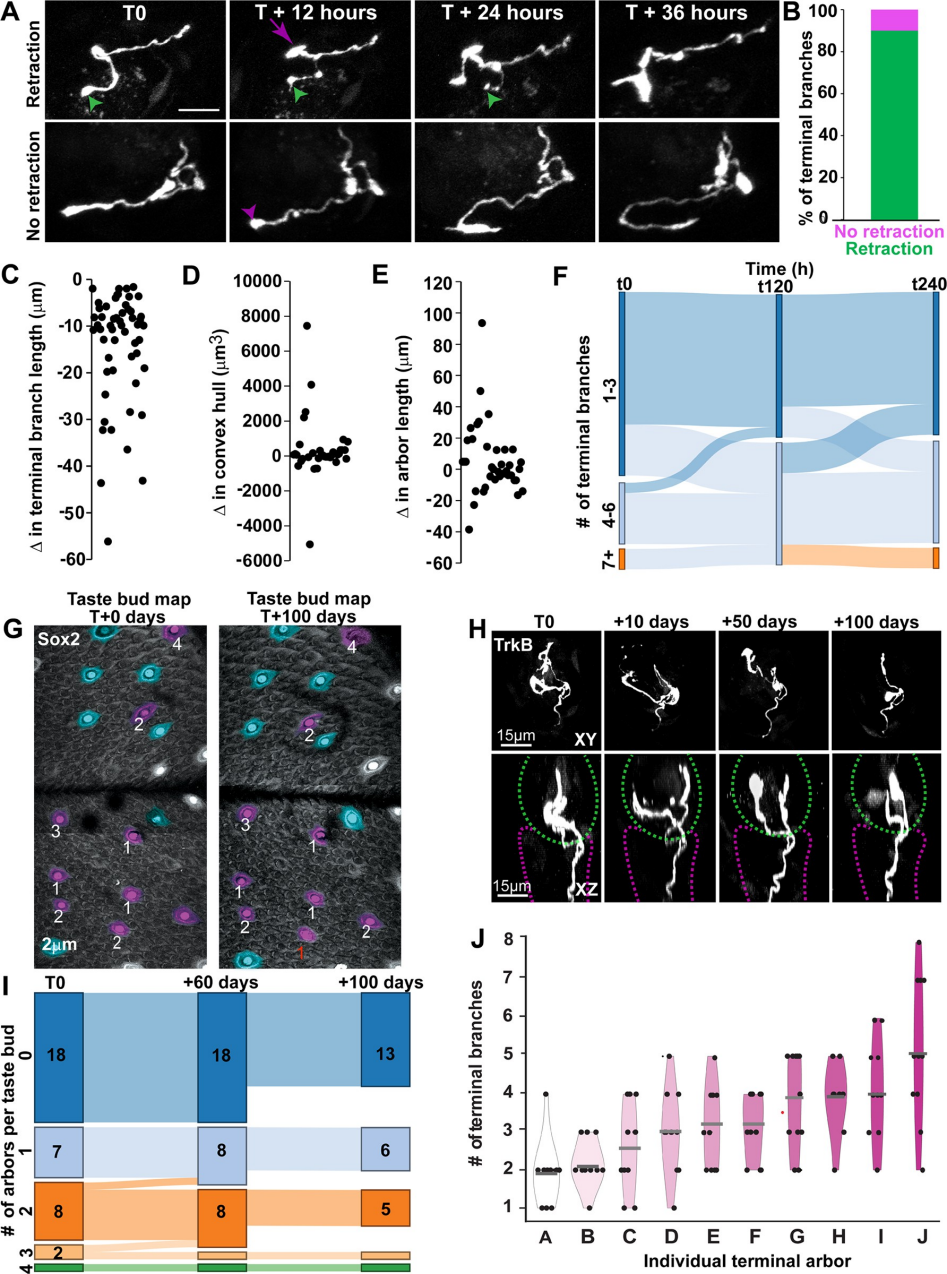
Retraction bulbs predict terminal branch retraction, and arbor number is stable over 100 days. (**A**) Two example arbors shown across a 36-hour window. In the top row, a terminal branch retracted after displaying a bulb at the tip of the ending (green arrowhead) and swellings within the branch. A separate terminal branch on this same arbor extended (magenta arrow) in conjunction with terminal branch retraction. In the bottom row, a terminal branch displayed an end-bulb and swellings but did not retract (magenta arrowhead); instead, it extended, indicating that the bulb may have been a growth cone. (**B**) Most terminal branches that displayed end-bulbs and swellings retracted at later time points (green); however, a small portion did not retract (magenta) (*n* = 12 arbors from 3 mice). (**C**) Change in terminal branch length. (**D**) Change in convex hull after each terminal branch retraction. (**E**) Net change in total arbor length after each terminal branch retraction. (**F**) Sankey diagram showing arbors grouped based on total number of terminal branches (1 to 3 = dark blue, 4 to 6 = light blue, 7+ = orange). Most arbors did not change groups at 5 (t120) or 10 (t240) days of imaging, demonstrating some stability in arbor complexity over time. (**G**) Two papillae maps of the same regions shown 100 days apart. Purple taste buds contained labeled arbors (number of arbors labeled in white), and blue taste buds did not contain labeled arbors. One papilla in this map lost an arbor during the 100-day window (red label). (**H**) Example arbor at days 0, 10, 50, and 100. The taste bud as defined by GFP is outlined in a green dashed line and papillae core in magenta. (**I**) Taste buds were categorized by number of labeled arbors and plotted in a Sankey diagram. No taste buds acquired new arbors, and 2 taste buds lost an arbor during the first 60 days (*n* = 3). One mouse was not imaged past day 60, so fewer taste buds were imaged between days 60 and 100 (*n* = 2); however, there was no loss of taste buds. (**J**) Violin plots of terminal branch number for 10 arbors imaged every 10 days for 100 days. Median indicated with gray bars. The data underlying the graphs in the figure can be found in https://data.mendeley.com/datasets/d58vz7wfrf/1.

Some terminal branches displayed distinct morphologies such as swellings on the tip of the terminal branch, as well as punctate swellings along the axon (Fig 4A). We suspected that these features could be associated with remodeling, as both retraction bulbs and growth cones have been described in taste arbors [9]. To determine if terminal branch swellings and beading were associated with gain or loss of terminal branches, we examined 12 arbors from 3 mice that were imaged every 12 hours for 10 days (*n* = 252 total time points). We observed 61 instances of swellings at the tip of the terminal branch and/or swelling along the branch. These terminal swellings along the axon were followed by retraction of the branch 90% of the time, whereas only 10% of terminal branches displayed terminal branch swelling without retracting (Fig 4B). The amount of retraction that occurred varied (Fig 4C) and took anywhere from 12 to 108 hours. Therefore, we conclude that terminal end swellings are often associated with retracting terminal branches (Fig 4A).

Since not all branch loss is associated with a retraction bulb, one possibility is that only those retractions with a retraction bulb occur when taste bud cells are lost. To determine if this was the case, we calculated the frequency of retractions that were associated with retraction bulbs. Retractions with retraction bulbs occurred once every 2 days on average for each arbor, which was also faster than the rate predicted by cell turnover (10 days). This finding indicates that branches retract for reasons other than loss of taste bud cells.

We next investigated whether retractions associated with retraction bulbs contributed to reduced arbor size using 2 measures of size before and after terminal branch retraction. The first measure was the smallest amount of 3D space enclosing the segmented arbor, which is the space occupied by the arbor within the taste bud (convex hull; Fig 4D). Physiologically, it is indicative of how many taste bud cells the arbor can reach. The second is the total length of the arbor (Fig 4E). Retraction of a single terminal branch did not alter total arbor size (Student unpaired *t* test, t = 0.4515, df = 74, *p* = 0.65). These lost branches may represent a small percentage of the total branch length. Consistently, the average length of retraction for a single branch identified using end bulbs and beading was 14.1 ± 11.9 μm (Fig 4C), which was a small portion of the total length of these arbors (mean = 79.2 ± 40.8 μm). In addition, branch formation/elongation commonly occurred in the arbor at different locations concurrently with terminal branch retraction. Consistently, 21 of 38 arbors increased in total length while retracting a terminal branch. These opposing changes in terminal branch length maintain arbor size over time.

Given that arbors add and lose terminal branches concurrently, arbor complexity over time may remain constant. To examine this possibility, we grouped arbors based on their complexity and examined their extent of change over 10 days. Because the simplest arbors typically showed a range of 3 terminal branches (Fig 2C), we grouped them accordingly. We observed that most arbors maintained a limited range of complexity over 10 days (Fig 4F), such that the minimum and maximum number of terminal branches for each arbor was correlated (R^2^ = 0.698, *p* < 0.001). Arbors from the simple group (1 to 3 branches) never remodeled to become complex arbors (more than 6 branches) within a 10-day imaging window. Although branch retractions occurred frequently within 10 days, they occurred simultaneously with terminal branch formation, which limited the range of complexity for most arbors.

### Variability in arbor size over 10 days differs across arbors

Because the volume occupied by the arbor within a taste bud (convex hull) was not heavily impacted by the retraction of single terminal branches, we hypothesized that arbor size is maintained despite constant terminal branch remodeling. To determine if this was the case, we examined whether arbor size (convex hull and total arbor length) changed over 10 days. To perform comparisons across all time points within a 10-day period, we developed a semiautomated image analysis pipeline in MATLAB, called ArborTools, to segment arbors and quantify convex hull and total arbor length (S3A Fig). Reconstructions of each arbor were verified visually to confirm that the arbor and the reconstruction matched (S3A Fig, cyan versus white). Arbors with too much background for automatic segmentation were removed from analysis. The volume occupied by the arbor (convex hull) changed in the taste bud across 10 days. In general, convex hull changed along with total length (S3A Fig). These changes in size could be due to changes in height, x or y width, or combinations of these changes (S3C Fig). For example, in the illustrated arbor (S3A-S3C Fig), increases in size occur due to an increase in Y width from 0 to 12 hours, and, dramatically, increases in size between hour 228 and 240 occur due to concurrent increases in X, Y width and height. When examining the extent to which individual arbors displayed changes in size, we noticed that some arbors appear to be more variable than others (S3D and S3E Fig).

### Number of arbors within a taste bud is stable over 100 days

We did not observe any new arbors growing into taste buds, and only a small number of arbors were lost within 10 days. Therefore, total number of arbors may be a stable feature for a given taste ganglion neuron over time. Alternatively, gain or loss of whole arbors may occur but could require more than 10 days. To distinguish between these possibilities, we imaged taste buds every 10 days for 60 or 100 days in mice where neurons were sparsely labeled. We observed taste buds with labeled arbors and taste buds without labeled arbors (*n* = 18 taste buds in 3 mice for 60 days, *n* = 13 taste buds in 2 mice for 100 days). Taste buds appeared to contain a stable number of arbors over 100 days; none were added and very few were lost (Fig 4G). The structure of the axon in the papillae core was also largely unchanged over 100 days (Fig 4H, purple dashed line). In addition, no taste buds acquired new labeled arbors in 60 or 100 days of imaging. Initially, 18 taste buds contained a total of 33 labeled arbors. After 60 days of imaging, 31 arbors were still present. No additional labeled arbors were lost from the remaining taste buds in the 2 mice imaged from 60 to 100 days (Fig 4I). As expected, these arbors remodeled over the 100 days of imaging (Fig 4J). These results suggest that the number of arbors is a stable feature of the neuron in adulthood.

## Discussion

Taste bud cells are continuously replaced through adulthood [1–3], but whether taste ganglion neurons remodel while reconnecting with new taste bud cells was unknown. In other systems, intravital imaging has been used to examine neuron remodeling during development, following injury, and in some cases during adulthood [15,18–21,26,27,44–47]. Here, we show that all peripheral taste axons have arbors that continuously remodel and, compared to plasticity in other systems, do so with unprecedented speed. For example, a structure well studied for its remodeling capabilities in adulthood, the dendritic spine, remodels over days [45], and at least half of the observed spines are stable over months [24,26]. In contrast to dendritic spines, some terminal branches of taste arbors appear and leave over much shorter timescales—sometimes lasting less than 12 hours. The speed and extent of remodeling in peripheral taste axons provides insight into 2 basic questions. First, how do arbors locate a new taste bud cell during the process of taste cell renewal? Secondly, is morphological variation across individual peripheral axons due entirely to structural plasticity, or can taste neurons be classified as distinct types based on anatomical differences? We discuss each of these issues below.

### The relationship between taste arbor remodeling and cell renewal

Most taste arbors only connect to 1 taste bud cell at any given time [10,11]. In a 10-day period, roughly half of the population of taste bud cells are lost [1]. Therefore, we anticipated that approximately half of all arbors sampled would lose their connection to a taste bud cell and/or gain a new connection within 10 days. We expected that arbor structure would be stable while connected to a taste bud cell, remodeling only when connections were broken [12]. This type of phasic remodeling is observed in terminations of touch neurons that receive input from Merkel cells [48]. The results of our study do not support the model that taste neurons experience distinct phases of remodeling and stability. Rather, the arbors of taste ganglion neurons continuously remodel with concurrent terminal branch growth and retraction, similar to the pattern of remodeling in axon terminals of ear-skin [46].

While the median life span of a taste bud cell is 10 to 12 days [1], different types of taste bud cells within the taste bud turnover at differing rates [3,4]. Since some arbors are likely to connect to 1 taste bud cell type and other neuron arbors to a different taste bud cell type, it might be expected that some arbors remodel faster than others. However, it is not clear that this is the case. All arbors showed at least some remodeling within 4 days. We did observe that terminal branch remodeling occurs to a greater extent in some arbors compared to others. It is possible that these arbors connect with cells having shorter life span. Yet, the rate of loss and gain of terminal branches, which required imaging every 4 hours to detect, occurs faster than the rate of cell turnover for cell types with the shortest life spans. Therefore, it seems unlikely that the faster rate of turnover in short-lived cells explains the rate at which terminal branches are gained and lost.

During remodeling, the arbors of taste ganglion neurons display similar anatomical features to motor axon terminals, which innervate neuromuscular junctions, and undergo remodeling as refinement occurs during development. However, refinement in motor axons largely consists of terminal branch retraction and pruning [18,23]. Branch retraction in motor axons is typically accompanied by retraction bulbs and terminal branch swellings. In addition to refinement, these features are also seen during degeneration after injury [22,23,49]. Similarly, the arbors of taste ganglion neurons have also been described as having these irregular swellings and end-bulbs [9]. Here, we found that branches displaying these features are primarily retracting. These observations suggest that retraction occurs by a conserved mechanism across systems—a process that involves axosomal vesicle shedding and engulfment of neuronal debris by phagocytic cells [23]. In the case of motor neurons, there is evidence that factors provided from the neuromuscular junction trigger terminal branch retraction [22]. While the anticipated source of such a signal in the taste bud would be from a dying taste bud cell, retraction occurs more frequently than would be predicted based on the rate at which cells are leaving the taste bud. Specifically, we observed 55 retractions preceded by retraction bulbs in 12 arbors over 10 days, if these retractions only occurred in response to the death of a connected cell, we would expect fewer than 1 retraction per arbor in 10 days. The frequency of terminal branch retractions suggests that a signal from a dying taste bud cell is not required to trigger terminal branch retraction in taste neuron terminal arbors.

Taste bud cells release growth and guidance factors. As new cells enter the taste bud, they could release factors that stimulate branch formation and/or extension [7,50–55]. However, our finding that terminal branch remodeling is much faster than expected based on the rate of cell turnover is not consistent with this possibility. Instead, terminal branch remodeling may be an intrinsic feature of taste ganglion neurons, occurring regardless of the external factors present. To assess whether this remodeling is driven by an intrinsic mechanism, we examined remodeling of terminal arbors while preventing addition of new cells into taste buds. Treatment with LDE225 inhibits taste bud cell differentiation such that new taste bud cells do not enter the taste bud [13,39]. Surprisingly, we found that preventing integration of new taste bud cells does not influence the frequency of terminal branch loss or formation.

Although taste receptor cell turnover does not influence terminal branch remodeling, it could be that neural activity regulates arbor plasticity. In fact, this is the case with the central terminations of these same axons [56,57]. However, we found that taste arbors remain and continue to remodel even after the entire taste bud is lost. These remaining afferents in fungiform papillae without taste buds, following Hh-pathway inhibition, were hypothesized to be the perigemmal touch-sensitive neurons from the geniculate ganglion rather than gustatory neurons [8]. However, using longitudinal imaging, we could examine the same arbors before and after Hh-pathway inhibition. We found that the majority of TrkB+ arbors remaining in the papillae after Hh-pathway inhibition were originally intragemmal. In addition, TrkB is not expressed by many perigemmal geniculate afferents [14]. Therefore, we conclude that these remaining arbors following Hh-signaling inhibition include taste arbors that no longer respond to taste stimuli [8], because taste-transducing cells are absent. Since these axons are no longer taste responsive, any remaining remodeling would not be influenced by taste activity. These remaining arbors contain growth-associated protein 43 (GAP43) [8], which is consistent with our finding that they continue to remodel even in the absence of a taste bud. Collectively, these findings support the conclusion that axonal arbor remodeling is not regulated by taste receptor cell turnover or taste activity, so it may be an intrinsic characteristic of peripheral taste neurons. However, it is also possible that remaining epithelial cells can influence remodeling.

Previously, we hypothesized taste arbors remodel to connect with new taste cells over time [12]. This model assumed that taste bud cells provide an extrinsic signal to influence arbor remodeling. Because this concept is not supported experimentally by the current study, we offer an updated model of how taste nerve fibers connect to new taste bud cells over time. We propose that arbors of taste ganglion neurons continuously sample the taste bud compartment by extending and retracting terminal branches, and this remodeling is orchestrated by an intrinsic cellular mechanism. An intrinsic program that drives constant remodeling allows taste arbors to detect extracellular signals and compare spatial arrangements of taste cells in their immediate environment. By extending branches randomly and continuously, nerve arbors eventually come near to a cell of the “correct type.” Once it is in the vicinity of the new taste bud cell, trophic mechanisms may attract the taste arbor a short distance to the cell. Furthermore, if constant terminal branch remodeling causes an arbor to contact a cell, then detection of surface molecules, such as protocadherins, could regulate the connection, and diffusible growth factors may be unnecessary [5]. In this scenario, terminal branch remodeling orchestrates the detection of extrinsic molecular cues, suggesting that remodeling is not a response by peripheral taste neurons to extrinsic molecular cues.

### The role of plasticity in individual peripheral taste axon morphology

A primary goal of the current study was to determine which structural features of peripheral taste neurons are plastic and which features are stable. Addressing this question is fundamental to determining whether taste neurons can be classified morphologically into separate types [58,59]. Taste neurons are pseudo-unipolar sensory neurons with a single axon and a primary bifurcation that creates a central and peripheral projection [60,61]. We recently described the morphology of the peripheral axons of taste neurons for the first time using a fixed-tissue approach [14]. The peripheral axons of taste neurons vary considerably in structure due to differences in the number of branch points within the tongue muscle, the lamina propria, and the taste bud [14]. Here, we show that taste arbors remodel, and individual arbors differ in terminal branch number because of structural plasticity.

Although terminal branches remodel quickly, the gain and loss of terminal branches occur concurrently, such that loss of a terminal branch does not always reflect a decrease in arbor size. Individual arbor size does vary over time; however, some individual arbors appear to change in size more than others. We recently found that individual taste neurons either have all small arbors or have a mix of large and small arbors [12]. So, it may be that arbor size only varies within a range of possibilities, particularly for small arbors. This range in possibilities in arbor size may be determined by some intrinsic feature of the neuron, for example, genetic types or differing functional characteristics.

Interestingly, the number of arbors labeled remained stable over 100 days. The presence or absence of arbors in the bud is dictated by branch points within the axon that are in the muscle or lamina propria. Because whole arbors were not added or lost, the branch points producing these arbors are not added and/or lost during adulthood. Even when taste buds are eliminated, many individual arbors remain in the location previously occupied by taste buds. The axons of taste ganglion neurons may have between 1 to 14 arbors [14]. Given that most arbors only connect to 1 to 2 taste bud cells, the number of arbors is the primary anatomical feature that determines how many taste bud cells provide input to a given neuron [11,14]. Neurons with more arbors are predicted to be both more sensitive and broadly tuned than neurons with fewer arbors [62]. Although there is evidence that peripheral taste neurons can change function over time [63], there is also likely some level of functional stability so that taste information is relayed to the brain as an interpretable code. Maintaining a constant number of arbors is one mechanism by which taste neurons could maintain functional stability over time.

Here, we determined that some anatomical features of peripheral taste axons are highly plastic while others display long-term stability. Stable anatomical features of taste ganglion neurons that also vary across neurons could define morphological neuron types. These stable morphological differences may correspond to genetically defined neuron types [58,59] or functional differences [64–66]. However, it is still unclear whether peripheral taste neuron types are defined by a combination of expression, function, and stable morphological features [67]. By describing which portions of taste axons are plastic, and which are stable, we are now poised to determine if stable morphological features differ across genetic or functional types.

## Materials and methods

### Ethics statement

All procedures were approved by the University of Louisville Institutional Animal Care and Use Committee (protocol #22151). All animals were cared for in accordance with guidelines set by the United States Public Health Service Policy on the Humane Care and Use of Laboratory Animals and the National Institutes of Health Guide for the Care and Use of Laboratory Animals.

### Animals

*TrkB*^CreER^ mice (*Ntrk2*^*tm3*.*1(cre/ERT2)Ddg*^;; ISMR catalog #JAX:027214, RRID:IMSR_JAX:027214) were crossed with Cre-dependent tdTomato mice [68]; RRID: IMSR_JAX:007914) to obtain *TrkB*^CreER^:tdTomato mice in which tdTomato is expressed following TrkB-driven Cre-mediated gene recombination. These mice were crossed with a Sox2^GFP^ line (Sox2^tm2Hoch^; ISMR catalog #JAX:017592, RRID:IMSR_JAX:017592). Phox2b-Cre mice (MRRC_034613-UCD) were also bred with tdTomato reporter mice for fixed tissue experiments. K14^CreERT^ mice; ISMR catalog #JAX:005107, RRID:IMSR_JAX:005107) were crossed with tdTomato reporter mice for lineage tracing of taste cells. For all experiments, mice were postnatal day 60 or older at the initial imaging session. Males and females were used across all in vivo experiments (24 males and 19 females in total). Mice were divided across experiments as follows: 10-day imaging (*n* = 5), 100-day imaging (*n* = 3), 4-hour imaging (*n* = 5), 1-hour imaging (*n* = 4), 6-hour imaging (*n* = 2), 4-hour live imaging Hh-inhibition (*n* = 11; vehicle *n* = 5, LDE225 *n* = 6), new cells entering the taste bud (*n* = 7; vehicle *n* = 2, LDE225 *n* = 5), confocal imaging experiments examining taste bud size (*n* = 10; vehicle *n* = 5, LDE225 *n* = 5).

### Tamoxifen administration

Tamoxifen (T5648, MiliporeSigma, St. Louis, MO) was dissolved in corn oil (C-8267, MiliporeSigma) at 20 mg/ml by shaking and heating at 42°C and injected in a single dose on postnatal day 40 by intragastric gavage. Mice were injected with 1.5 mg of tamoxifen to label a subset of arbors.

### LDE225 administration

Mice were treated by daily oral gavage for 10 days with vehicle (PEG 400:5% dextrose in water) or LDE225 (NVP-LDE225, ChemieTek, Indianapolis, IN) dissolved in vehicle at a dose of 20 mg/kg. The dose was determined based on [13] and preliminary studies. The oral gavage probe (22-gauge, 25 mm; Instech) by-passed all oral tissues for direct delivery into the stomach.

### Immunohistochemistry

Phox2b-Cre:tdTomato mice were killed by overdose of tribromoethanol (avertin, 4 mg/kg) and perfused transcardially with 4% paraformaldehyde. Dissected tissues were postfixed in 4% paraformaldehyde for 2 hours (for thin serial sections) or overnight (thick sections and whole mounts), rinsed with PBS, and transferred to 30% sucrose at 4°C overnight. A razor blade was used to remove the anterior two-thirds of the tongue, after which tongues were carefully split down the midline with a razor blade under a dissection microscope. Tongues were frozen the next day in OCT and stored at −80°C before processing for whole-mount staining. Whole-mount immunohistochemistry of the lingual epithelium was performed to visualize innervated taste buds. First, the underlying muscle and lamina propria were removed as described previously [69]. The isolated lingual epithelium was then washed for 15 minutes (3 times) in 0.1 M PBS. Tissues were then incubated in blocking solution (3% donkey serum and 0.5% Triton X-100 in 0.1 M PBS) at 4°C overnight and then incubated for 5 days at 4°C with primary antibodies (DsRed (rabbit) and Troma1 (rat)) in antibody solution (0.5% Triton X-100 in 0.1 M PBS). Tissues were rinsed 4 times for 15 minutes each with 0.1 M PBS and incubated with secondary antibodies anti-DsRed (1:500; RRID:AB_10013483; Living Colors DsRed polyclonal; Takara Bio USA, San Jose, CA) and anti-Troma1 (1:500, Alexa Fluor 488 AffiniPure, RRID:AB_2340619; Jackson ImmunoResearch, West Grove, PA). Tissues were then rinsed again, mounted with Fluoromount-G, and coverslipped (high precision, 0107242; Marienfeld).

### Confocal imaging

Taste bud images were obtained using an Olympus Fluoview FV1000 confocal laser-scanning microscope with a 60× NA1.4 lens objective using a zoom of 3, Kalman 2. Image sizes were initially set at 1,024 × 1,024 pixels but were cropped to reduce scanning time and bleaching. Serial optical sections at intervals of 0.47 μm in the Z dimension were captured, which is the optimal size at 60× magnification for 3D reconstruction. All colors were imaged sequentially in separate channels to avoid bleed-through. Image stacks were then deconvolved using AutoQuant X3 software (Media Cybernetics, Rockville, MD) to reduce out-of-focus fluorescence and improve image quality.

### Two-photon in vivo imaging

In vivo 2PLSM imaging was performed using a Movable Objective Microscope (Sutter Instruments, Novato, CA). A Fidelity-2 1,070-nm laser (Coherent, Silicon Valley) was used to visualize tdTomato, and a Chameleon tunable laser (Coherent) set to 920 nm was used to visualize GFP. The excitation wavelength at the sample ranged from 20 51 milliWatts measured at the exit of the microscope objective. A custom-built tongue holder, based on a published design, was used to stabilize the anterior tongue for imaging of the dorsal surface [70,71]. Mice were anesthetized using a 0.6% isoflurane (Henry Schien, Melville, NY) and O_2_ mixture. A temperature controller was used to monitor and maintain body temperature at 36°C. Ophthalmic lubricant ointment (Henry Schien) was applied to the eyes. Taste bud maps were acquired using a 10 × 0.3 numerical aperture water immersion objective lens (Carl Zeiss, USA), and images of arbors were acquired using a 40 × 1.0 numerical aperture water immersion lens (Zeiss). An early version of ScanImage (MATLAB) was used to collect images [72]. Arbors were typically imaged to 70 μm in depth below the surface of the tongue, and 80 × 80 μm optical sections (512 × 512 pixels) were collected at 0.5 or 1 μm increments.

### Image analysis

Taste bud volume was calculated by contouring whole-mount-stained taste buds at 3 μm increments perpendicular to the XY plane of the confocal z-stack. K14+ cells were manually quantified from taste bud z-stacks using ImageJ. Terminal branches of sparsely labeled receptive arbors were manually quantified from z-stacks using ImageJ.

Terminal branches that displayed swellings were quantified using the Image J plugin, SNT. Branch endings were identified as having swellings when the swellings were at least 3 times wider than the branch. Once all the swellings for 12 arbors over 10 days had been identified, each subsequent day was examined and the branch with the swelling was reconstructed to the nearest branch point. In instances where the branch was shorter at the second measurement (retraction), the branch continued to be measured every 12 hours until the branch stopped retracting or disappeared. The length of the branch at the final branch time point was subtracted from the length at the initial time point with the swelling to determine the total length of the retraction.

A custom MATLAB program (ArborTools, https://github.com/Dalston817/ArborTools) was used to automatically segment arbors. 3D images stacks were cropped to isolate terminal arbors from background. A custom filter was used to extract initial segmentation volumes [73]. After automatic segmentation, the segmented arbors were compared to the arbors and instances where background pixels were segmented were manually selected to not be included in the convex hull and length analysis. From segmented volumes, skeletons were extracted using the Treestoolbox [74]. Segmented arbors are available here (https://neuromorpho.org/dableFiles/whiddon_krimm/Supplementary/swc_files.zip). All convex hull and arbor length data reported were generated from ArborTools.

### Statistical analysis

Rates of terminal branch change were compared across image sets by first testing whether the measure was normally distributed using a Shapiro–Wilk test. If a measure was normally distributed, then differences were determined using a Student *t* test for 2 groups or one-way analysis of variance with a Tukey post hoc test for more than 2 groups. If a measure was not normally distributed, then differences were determined using a Mann–Whitney test for 2 groups or Kruskal–Wallis test for more than 2 groups. A *p*-value <0.05 was used to determine statistical significance.

## Supporting information

S1 Fig In vivo imaging of the mouse tongue. (**A**) The preparation used for intravital imaging of the mouse tongue. The imaging platform was made resembling that already published [70,71]. (**B**) Z-projection of a taste bud in the x-y plane in which the taste bud was imaged parallel to the tongue surface. (**C**) Y-projection of the image stack, assembled from the stack of individual images scanned at each μm. Scale bar = 10 μm and applies to (B) and (C).
(TIF)

S2 Fig LDE225 inhibits new cell entry into the taste bud, resulting in volume reduction by day 10. **(A-C)** A subset of epidermal basal cells expressing *K14*^CreER^:tdTomato were labeled with a single dose of tamoxifen immediately preceding 10 days of treatment with vehicle or LDE225. On day one, tdTomato label was restricted to the region where the K14+ basal cells wrap around the taste bud. The taste bud is defined with yellow dashed border. Over the 10 days of treatment, these cells entered the taste bud (cyan arrow) from the vehicle-treated (**A**) but not the LDE225-treated animal (**B**). LDE225-treated mice had fewer labeled cells in taste buds than vehicle-treated mice by day 10 (**C**). Specifically, 27 of 40 taste buds completely lacked new labeled cells following LDE225 treatment, while all those examined had some labeled cells following vehicle treatment. (**D-H**) Whole-mounts of the lingual epithelium were labeled for taste buds (green, keratin 8) and neurons (Phox2b-tdTomato, red) following 10-day of treatment with vehicle (**D,** z-plane; **E,** x-y plane) and LDE225 (**F,** z-plane; **G,** x-y plane) tongues. Scale bar = 10 μm and applies to (D-G). (**H**) Taste bud volumes were smaller following 10 days of LDE225 compared with vehicle controls. *p ≤ 0.05, ***p ≤ 0.001. The data underlying the graphs in the figure can be found in https://data.mendeley.com/datasets/d58vz7wfrf/1.
(TIF)

S3 Fig Taste arbors vary in size over 10 days. A custom image analysis pipeline was developed to segment arbor structure from raw image TIF stacks. (**A**) Examples of convex hulls (purple) and arbor skeletons (cyan) generated from segmentation data are shown in 2 orientations and at 3 time points. Colored asterisks at each time point are used to indicate convex hull and arbor length in (C). (**B**) Convex hull size (purple line) and arbor length (cyan) for the example arbor in (A) are shown across the entire 10-day imaging window. **(C)** Convex hull potted with z-height, x-width, and y-width over time. (**D**) Convex hull and (**E**) arbor length measured for 13 individual arbors arranged by ascending convex hull. Means are indicated with red lines. The data underlying the graphs in the figure can be found in https://data.mendeley.com/datasets/d58vz7wfrf/1.
(TIF)

S1 Movie The arbor from Fig 3A (X-Y view) from T0-T228, every 12 hours = 0.5 seconds. Time in hours is on the bottom right-hand side of the images. Branches are added and lost to the portion of the arbor inside the taste bud.
(MP4)
