# Supplementary figures and images for: Rapid structural remodeling of peripheral taste neurons is independent of taste cell turnover
Source: PLoS Biol. 2023 Aug 31;21(8):e3002271. doi: 10.1371/journal.pbio.3002271 (PMC10499261; doi:10.1371/journal.pbio.3002271)

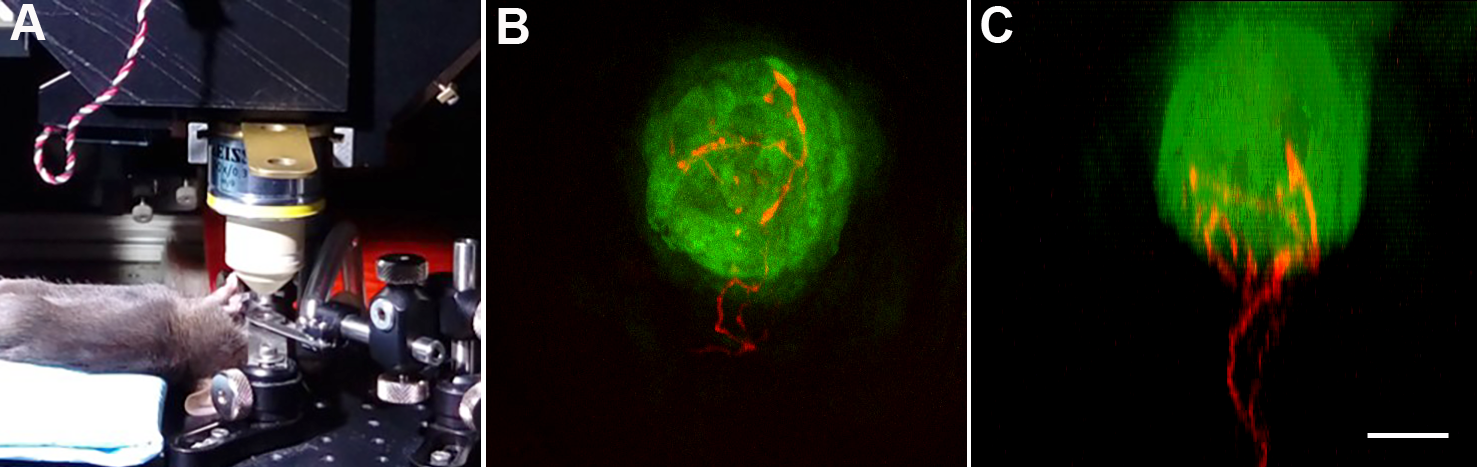

Supplement: S1 Fig — (A) The preparation used for intravital imaging of the mouse tongue. The imaging platform was made resembling that already published [70,71]. (B) Z-projection of a taste bud in the x-y plane in which the taste bud was imaged parallel to the tongue surface. (C) Y-projection of the image stack, assembled from the stack of individual images scanned at each μm. Scale bar = 10 μm and applies to (B) and (C). (TIF) [file pbio.3002271.s001.tif]

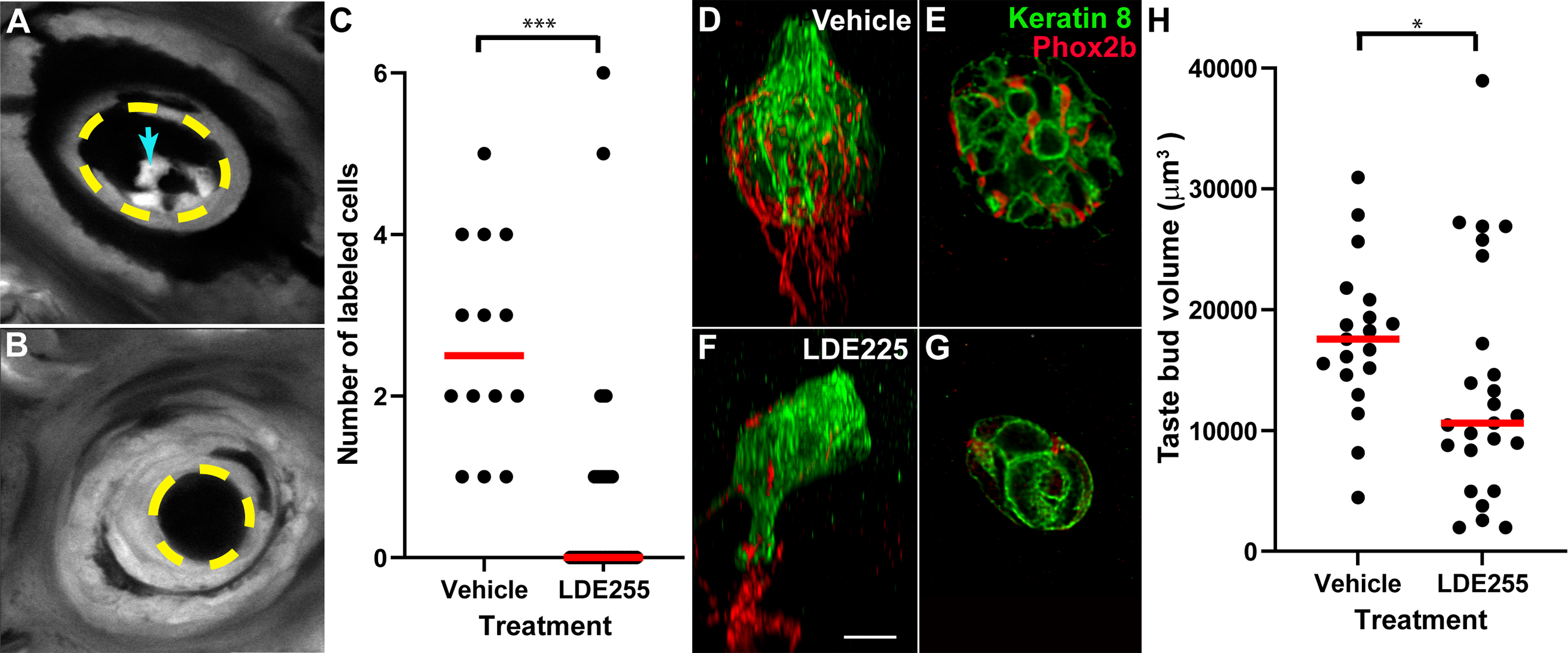

Supplement: S2 Fig — (A-C) A subset of epidermal basal cells expressing K14CreER:tdTomato were labeled with a single dose of tamoxifen immediately preceding 10 days of treatment with vehicle or LDE225. On day one, tdTomato label was restricted to the region where the K14+ basal cells wrap around the taste bud. The taste bud is defined with yellow dashed border. Over the 10 days of treatment, these cells entered the taste bud (cyan arrow) from the vehicle-treated (A) but not the LDE225-treated animal (B). LDE225-treated mice had fewer labeled cells in taste buds than vehicle-treated mice by day 10 (C). Specifically, 27 of 40 taste buds completely lacked new labeled cells following LDE225 treatment, while all those examined had some labeled cells following vehicle treatment. (D-H) Whole-mounts of the lingual epithelium were labeled for taste buds (green, keratin 8) and neurons (Phox2b-tdTomato, red) following 10-day of treatment with vehicle (D, z-plane; E, x-y plane) and LDE225 (F, z-plane; G, x-y plane) tongues. Scale bar = 10 μm and applies to (D-G). (H) Taste bud volumes were smaller following 10 days of LDE225 compared with vehicle controls. *p ≤ 0.05, ***p ≤ 0.001. The data underlying the graphs in the figure can be found in https://data.mendeley.com/datasets/d58vz7wfrf/1. (TIF) [file pbio.3002271.s002.tif]

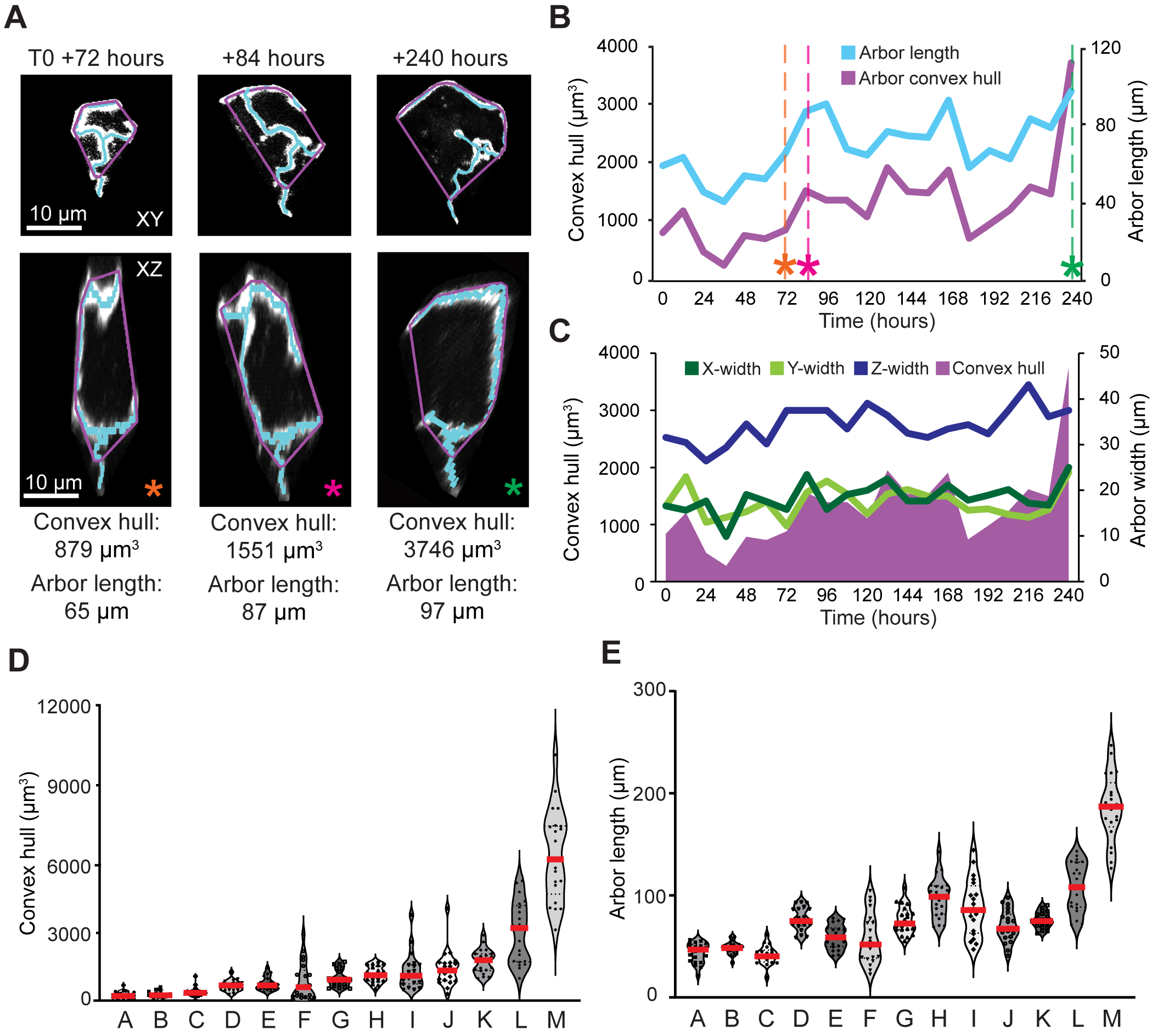

Supplement: S3 Fig — A custom image analysis pipeline was developed to segment arbor structure from raw image TIF stacks. (A) Examples of convex hulls (purple) and arbor skeletons (cyan) generated from segmentation data are shown in 2 orientations and at 3 time points. Colored asterisks at each time point are used to indicate convex hull and arbor length in (C). (B) Convex hull size (purple line) and arbor length (cyan) for the example arbor in (A) are shown across the entire 10-day imaging window. (C) Convex hull potted with z-height, x-width, and y-width over time. (D) Convex hull and (E) arbor length measured for 13 individual arbors arranged by ascending convex hull. Means are indicated with red lines. The data underlying the graphs in the figure can be found in https://data.mendeley.com/datasets/d58vz7wfrf/1. (TIF) [file pbio.3002271.s003.tif]
